# Inhibition of RNA polymerase II allows controlled mobilisation of retrotransposons for plant breeding

**DOI:** 10.1186/s13059-017-1265-4

**Published:** 2017-07-07

**Authors:** Michael Thieme, Sophie Lanciano, Sandrine Balzergue, Nicolas Daccord, Marie Mirouze, Etienne Bucher

**Affiliations:** 1 Botanical Institute, Zürich-Basel Plant Science Center, University of Basel, Hebelstrasse 1, 4056 Basel, Switzerland; 2 Institut de Recherche pour le Développement, UMR232 DIADE Diversité Adaptation et Développement des Plantes, Université Montpellier 2, Montpellier, France; 3 University of Perpignan, Laboratory of Plant Genome and Development, 58 Avenue Paul Alduy, 66860 Perpignan, France; 4 IRHS, Université d’Angers, INRA, AGROCAMPUS-Ouest, SFR4207 QUASAV, Université Bretagne Loire, 49045 Angers, France

**Keywords:** Epigenetics, DNA methylation, Genome integrity, Evolution, *Oryza sativa*, *Arabidopsis thaliana*

## Abstract

**Background:**

Retrotransposons play a central role in plant evolution and could be a powerful endogenous source of genetic and epigenetic variability for crop breeding. To ensure genome integrity several silencing mechanisms have evolved to repress retrotransposon mobility. Even though retrotransposons fully depend on transcriptional activity of the host RNA polymerase II (Pol II) for their mobility, it was so far unclear whether Pol II is directly involved in repressing their activity.

**Results:**

Here we show that plants defective in Pol II activity lose DNA methylation at repeat sequences and produce more extrachromosomal retrotransposon DNA upon stress in *Arabidopsis* and rice. We demonstrate that combined inhibition of both DNA methylation and Pol II activity leads to a strong stress-dependent mobilization of the heat responsive *ONSEN* retrotransposon in *Arabidopsis* seedlings. The progenies of these treated plants contain up to 75 new *ONSEN* insertions in their genome which are stably inherited over three generations of selfing. Repeated application of heat stress in progeny plants containing increased numbers of *ONSEN* copies does not result in increased activation of this transposon compared to control lines. Progenies with additional *ONSEN* copies show a broad panel of environment-dependent phenotypic diversity.

**Conclusions:**

We demonstrate that Pol II acts at the root of transposon silencing. This is important because it suggests that Pol II can regulate the speed of plant evolution by fine-tuning the amplitude of transposon mobility. Our findings show that it is now possible to study induced transposon bursts in plants and unlock their use to induce epigenetic and genetic diversity for crop breeding.

**Electronic supplementary material:**

The online version of this article (doi:10.1186/s13059-017-1265-4) contains supplementary material, which is available to authorized users.

## Background

Like retroviruses, long terminal repeat (LTR) retrotransposons (class I elements), which represent the most abundant class of transposable elements (TEs) in eukaryotes, transpose via a copy and paste mechanism. This process requires the conversion of a full length RNA polymerase II (Pol II) transcript into extrachromosomal complementary DNA (ecDNA) by reverse transcription [1]. In their life cycle LTR retrotransposons can produce extrachromosomal circular DNA (eccDNA), which is an indicator for their ongoing activity [2]. In plants, TEs are increasingly seen as a source of genetic and epigenetic variability and thus important drivers of evolution [3–6]. However, plants have evolved several regulatory pathways to retain control over the activity of these potentially harmful mobile genetic elements. Cytosine methylation (^m^C) plays a central role in TE silencing in plants [7]. In addition, plants have evolved two Pol II-related RNA polymerases, Pol IV and Pol V, that are essential to provide specific silencing signals leading to RNA-directed DNA methylation (RdDM) at TEs [8], thereby limiting their mobility [9–11]. More recently, various additional non-canonical Pol IV-independent RdDM pathways have been described [12]. Notably it was found that Pol II itself also plays an important role in RdDM [13, 14] by feeding template RNAs into downstream factors such as RNA-DEPENDENT RNA POLYMERASE 6 (RDR6), resulting in dicer-dependent or -independent initiation and establishment of TE-specific DNA methylation [15]. Beyond that, recent work suggests a new “non-canonical” branch of RdDM that specializes in targeting transcriptionally active full-length TEs [16]. This pathway functions independently of RDRs via Pol II transcripts that are directly processed by DCL3 into small interfering RNAs (siRNAs).

## Results

**Fig. 1:**
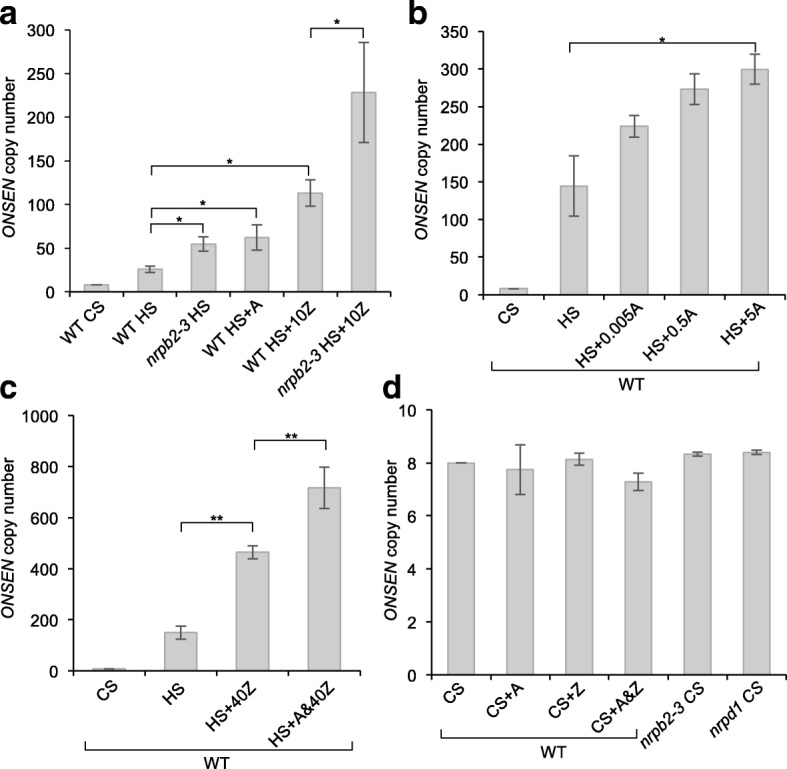
Pol II represses the HS-dependent mobility of the *ONSEN* retrotransposon in *Arabidopsis. ONSEN* copy number in *Arabidopsis* seedlings measured by qPCR directly after CS and HS treatments. **a** In the WT and the *nrpb2-3* mutant and after HS plus treatments with α-amanitin (*A*; 5 μg/ml) or zebularine (*Z*; 10 μM) (mean ± standard error of the mean (s.e.m.), *n* = 6 biological repetitions). **b** In the WT and after HS plus treatment with A at different concentrations (μg/ml) as specified on the x-axis (mean ± s.e.m., *n* = 4 biological repetitions). **c** In the WT and after HS plus treatment with Z (40 μM) or a combination of A (5 μg/ml) and Z (*A&40Z*) (mean ± s.e.m., *n* = 3 biological repetitions). **d** In the WT after chemical treatment with A (5 μg/ml), Z (40 μM), a combination of A and Z (*A&Z*) or in the *nrpb2-3* and *nrpd1* backgrounds following CS (mean ± s.e.m., *n* = 3 biological repetitions). All values are relative to *ACTIN2*. *P < 0.05, **P < 0.01

Here, we wanted to investigate if Pol II could play a direct role in repressing TE mobility in plants. For this purpose we chose the well-characterized heat-responsive *copia*-like *ONSEN* retrotransposon [11] of *Arabidopsis* and took advantage of the hypomorphic *nrpb2-3* mutant allele that causes reduced NRPB2 (the second-largest component of Pol II) protein levels [14]. Using quantitative real-time PCR (qPCR), we determined that challenging *nrpb2-3* seedlings by heat stress (HS) led to a mild increase in total *ONSEN* copy number (sum of ecDNA, eccDNA and new genomic insertions) relative to control stress (CS) and compared to the wild type (WT) (Fig. 1a). This result is supported by the observed dose-responsive increase in *ONSEN* copy number after HS and pharmacological inactivation of Pol II with α-amanitin (A), a potent Pol II inhibitor [17] that does not affect Pol IV or Pol V [18] (Fig. 1b). In order to test the interaction between Pol II-mediated repression of TE activation and DNA methylation, we grew WT and *nrpb2-3* plants on media supplemented with zebularine (Z), an inhibitor of DNA methyltransferases active in plants [19], and subjected them to HS. To ensure the viability of the *nrpb2-3* seedlings we choose a moderate amount of Z (10 μM). The presence of Z in the medium during HS generally enhanced the production of *ONSEN* copies. Importantly, this induced increase in *ONSEN* copy number was more distinct in the *nrpb2-3* background (Fig. 1a). This indicated that both DNA methylation and Pol II transcriptional activity contribute to the repression of *ONSEN* ecDNA production. To complete their lifecycle, the reverse transcribed ecDNA of activated retrotransposons has to integrate back into the genome [1]. Given that we observed a strong increase in *ONSEN* copy number after HS and treatment with moderate amounts of Z in the *nrpb2-3* background, we wanted to address the inheritance of additional *ONSEN* copies by the offspring. For this we compared the average *ONSEN* copy number of pooled S1 seedlings obtained from Z-treated and heat-stressed WT and *nrpb2-3* plants grown under controlled conditions on soil by qPCR. We observed a distinct increase in the overall *ONSEN* copy number exclusively in the *nrpb2-3* background (Additional file 1: Figure S1).

**Fig. 2:**
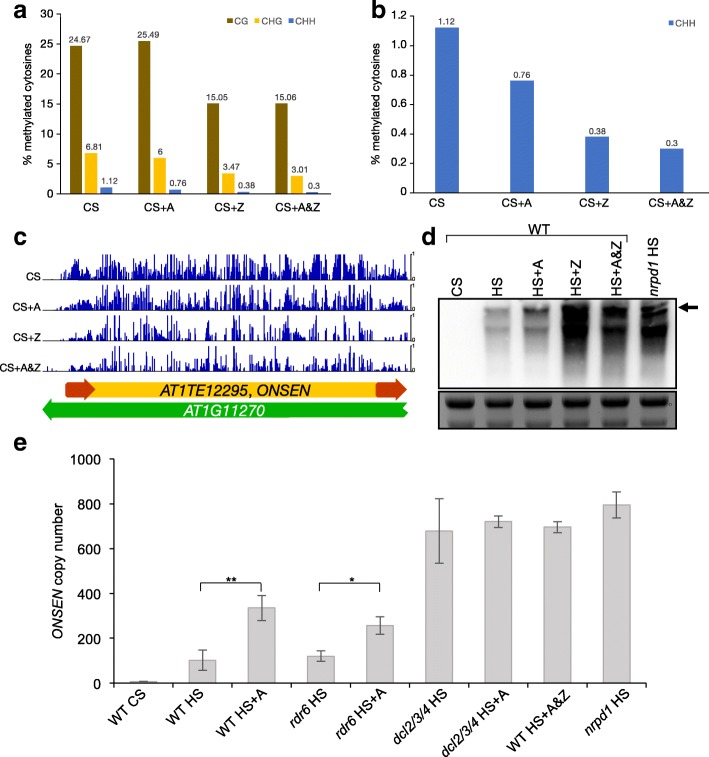
Simultaneous inhibition of DNA methyltransferases and Pol II reduces global CHH methylation and mimics the TE silencing deficiency of the *nrpd1* background. **a** Genome-wide DNA methylation levels in the WT after CS and CS plus treatment with A (5 μg/ml), Z (40 μM), or a combination of A and Z (*A&Z*) for three sequence contexts (*brown* for CG, *yellow* for CHG and *blue* for CHH). **b** Same as **a** but only depicting the CHH context for clarity. **c** Methylome data of treated and untreated plants at an *ONSEN* locus located on Chr 1 (*ONSEN* is indicated in *yellow*, its LTRs in *red*). **d** Northern blot of *ONSEN* transcripts directly after CS, HS and HS plus treatment with A, Z or a combination of A&Z in the WT and after HS in *nrpd1* plants. The *black arrow* indicates the *ONSEN* full-length transcript. Below, a Midori-stained agarose gel is shown as a loading control. **e** *ONSEN* copy number measured by qPCR directly after CS and HS treatments in WT, *rdr6*, *dcl2/3/4* and *nrpd1* seedlings directly after CS, HS and HS plus treatment with A, Z or a combination of A&Z (*mean ± s.e.m, n* = 3 biological repetitions, values relative to *ACTIN2*; *P < 0.05, **P < 0.01)

Because both DNA methylation and Pol II can be inhibited by the addition of specific drugs, we wanted to test if treating WT plants with both A and Z at the same time could strongly activate and even mobilize *ONSEN* after a HS treatment. We grew WT seedlings on MS medium supplemented with Z (40 μM) [19] individually or combined with A (5 μg/ml, A&Z). Consistent with the strong activation of *ONSEN* in HS and Z-treated *nrpb2-3* seedlings, the combined treatment (A&Z) of the WT gave rise to a very high (Fig. 1c) HS-dependent (Fig. 1d) increase in *ONSEN* copy number, comparable to that in the *nrpd1* background (Fig. 2e). We noted that the overall amplitude of HS-dependent *ONSEN* activation could vary between different waves of stress applications in terms of copy number (Fig. 1a, b). Yet, the observed enhancing effect of Pol II and DNA methyltransferase inhibition with A and Z on *ONSEN* activation was consistent in independent experiments (Figs. 1a–c and 2e). To detect activated TEs at the genome-wide level we took advantage of the production of eccDNA by active retrotransposons. eccDNA is a byproduct of the LTR retrotransposon life cycle [20]. Using mobilome sequencing, which comprises a specific amplification step of circular DNA followed by high-throughput sequencing to identify eccDNA derived from active LTR retrotransposons [2], we found that only *ONSEN* was activated by HS in combination with A&Z (Additional file 1: Figure S2). Confirming our qPCR data, more *ONSEN*-specific reads were detected in the presence of A and Z in the medium.

To better understand the mechanisms by which the drugs enhanced the activation of *ONSEN* after HS at the DNA level, we assessed how they influenced DNA methylation at the genome-wide level using whole-genome bisulfite sequencing (WGBS) after CS. Overall, we found that all drug treatments affected global DNA methylation levels. While the treatment with Z affected all sequence contexts, we observed that inhibition of Pol II primarily affected cytosine methylation in the CHG and CHH sequence contexts (where H is an A, T or G). The combined A&Z treatment had a slight additive de-methylating effect in the CHG and CHH contexts compared to A or Z alone (Fig. 2a, b). DNA methylation levels at one *ONSEN* locus (*AT1TE12295*) is depicted in Fig 2c. Treatment with A led to a slight decrease in DNA methylation, which was more apparent in Z- and A&Z-treated plants. We then checked by northern blot whether the degree of reduction in DNA methylation would coincide with increased *ONSEN* transcript levels directly after HS. We found that treatment with Z alone resulted in the highest *ONSEN* transcript level after HS (Fig. 2d). Considering the data obtained on *ONSEN* ecDNA (Fig. 1c), we concluded that a substantial proportion of these Z-induced transcripts were not suitable templates for *ONSEN* ecDNA synthesis.

In *Drosophila*, it has been shown that Pol II-mediated antisense transcription results in the production of TE-derived siRNAs in a Dicer-2-dependent manner [21]. In support of this in *Arabidopsis*, a recent publication pointed out the importance of DCL3 in regulating *ONSEN* in the *ddm1* background [16]. To elucidate whether the effect of Pol II inhibition was also dicer-dependent, we grew both *rdr6* and *dcl2/3/4* triple mutant plants on A, applied HS and measured *ONSEN* ecDNA levels. Strikingly, we found that A still enhanced ecDNA accumulation in *rdr6* plants, whereas inhibition of Pol II had no additional effect in the *dcl2/3/4* triple mutant (Fig. 2e).

**Fig. 3:**
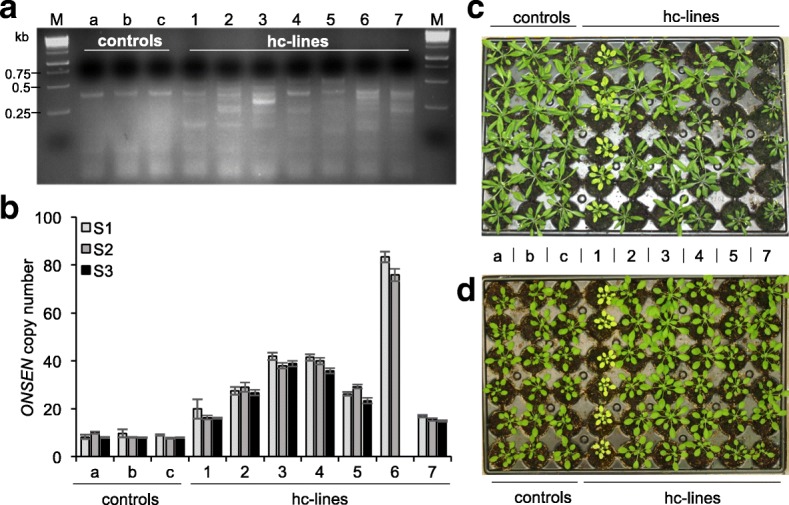
Drug-induced mobilization of *ONSEN* in WT *Arabidopsis* plants. **a** Transposon display testing seedlings in the S2 generation of WT plants for novel *ONSEN* insertions: lanes a to c show HS-treated plants; lanes 1 to 7 show hc lines 1–7 treated with HS and A (5 μg/ml) and Z (40 μM), M indicates the size marker. **b** *ONSEN* copy number in the S1, S2 and S3 generations measured by qPCR (mean ± s.e.m,* n* = 3 technical replicates, values relative to *ACTIN2*). **c**, **d** Photographs of S2 plants showing both homogeneous and environment-dependent phenotypic variability induced by the *ONSEN* mobilization when grown under long (**c**) and short day (**d**) conditions. qPCR data for the S3 generation of line 6 in **b** as well as pictures of phenotypes in **c** and **d** are missing due to severe infertility and extinction of this line

**Fig. 4:**
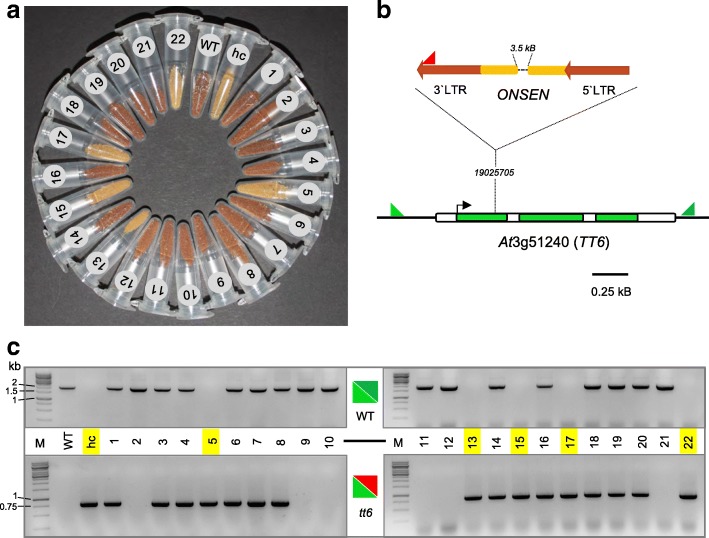
*Transparent testa* phenotype of hc line 3 co-segregates with an *ONSEN* insertion in *TT6*. Seed phenotypes (**a**) and corresponding genotypes (**c**) of a segregating F2 population (lanes 1–22) obtained from a cross between the WT and hc line 3 (*hc*) are shown. **b** Primers used for genotyping of the *ONSEN* insertion. For the WT-PCR depicted in the upper part of **c** the light (*tt6 fw*) and dark *(tt6 rev*) green primers flanking the *TT6* locus (*AT3G51240*) were used. The *ONSEN* insertion in *TT6* was detected by a combination of the light green primer with the red primer specific to the *ONSEN* LTR (*Copia* 78 3′ LTR, *red arrow*). M indicates the size marker. Primer sequences are given in Additional file 1: Table S1

Induced mobilization of endogenous TEs in plants has so far been very inefficient, thus limiting their use in basic research and plant breeding [3]. In the case of *Arabidopsis*, transposition of *ONSEN* in HS-treated WT plants has not been observed [11, 22]. Because the A&Z drug treatment resulted in high accumulation of *ONSEN* copy numbers—essentially mimicking plants defective in NRPD1 (Fig. 2e)—we wanted to test if the combined drug treatment could lead to efficient *ONSEN* mobilization in WT plants. First, we assessed by qPCR if, and at what frequencies, new *ONSEN* copies could be detected in the progeny of A&Z-treated and heat stressed plants. In fact, we found new *ONSEN* insertions in 29.4% of the tested S1 (selfed first generation) pools (n = 51), with pools having up to 52 insertions (Additional file 1: Figure S3). We then confirmed stable novel *ONSEN* insertions in a subset of independent individual high copy plants by transposon display (Fig. 3a), qPCR (Fig. 3b) and sequencing of 11 insertions in a selected high-copy line (hc line 3; Fig. 4; Additional file 1: Figure S4). Tracking *ONSEN* copy numbers over three generations of selfing indicated that the new insertions were stably inherited (Fig. 3b). Furthermore, the re-application of heat stress and drugs in the S3 generation of two hc lines did not lead to greater accumulation of *ONSEN* copies compared to control lines, but we instead observed stronger silencing in lines with more *ONSEN* copies (Additional file 1: Figure S5).

TE insertions can interrupt genes or alter their expression by recruiting epigenetic marks or by stress-dependent readout transcription from the 3′ LTR into flanking regions [6]. To test this, we grew the S2 generation of the selected hc lines under long- and short-day conditions. Interestingly, we observed that many hc lines showed clear and homogenous phenotypes in response to the different growth conditions (plant size, chlorophyll content and flowering time; Fig. 3c, d).

To demonstrate that *ONSEN* insertions could directly influence such developmental phenotypes, we closely investigated hc line 3, which produced white seeds (Fig. 4a). Using a candidate gene approach, we found that an *ONSEN* insertion in *transparent testa 6* (*TT6*, *AT3G51240*; Fig. 4b) was responsible for the recessive white seed phenotype [23, 24]. This was confirmed by segregation analysis of the F2 generation of a cross between WT and hc line 3 (Fig. 4a) followed by genotyping (Fig. 4c).

**Fig. 5:**
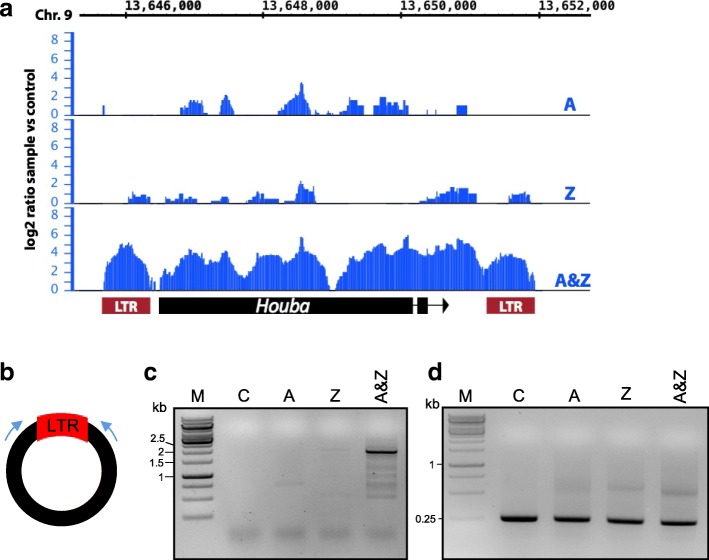
Drug-induced activation of the *Houba* retrotransposon in *O. sativa*. Mobilome analysis of DNA extracted from seedlings after growth under control conditions (*C*), A (5 μg/ml), Z (40 μM) or the combination of A&Z. **a** Logarithmic ratio of the depth of coverage obtained after aligning the sequenced reads on one *Houba* element. **b** Primer localization (*black bar*, *Houba* element; *arrows*, PCR primers; *red box*,LTR). **c** Circular forms of *Houba* are specifically detected in plants treated with A&Z using inverse PCR with primers shown in (**b**). **d** Specific PCR on chloroplast DNA is shown as a loading control. Total DNA subjected to a rolling circle amplification was used as a template. M indicates the size marker

Next, we wanted to test if Pol II plays a more general role in repressing TEs in plants. Due to its significantly different epigenetic and TE landscape compared to *Arabidopsis*, we wanted to test if we could mobilize TEs in rice (*Oryza sativa*) [25], a genetically well-characterized monocotyledonous crop. To capture drug-induced mobilized TEs, we characterized the active mobilome in *O. sativa* seedlings that were grown on MS medium supplemented with no drugs, A only, Z only or a combination of A and Z, using the same approach as we used for *Arabidopsis*. We identified *Houba*, a copia-like retrotransposon [26], as highly activated only when plants were treated with A&Z (Fig. 5a). Bona fide activity of *Houba* was supported by the detection of eccDNA containing LTR–LTR junctions (Additional file 1: Figure S6). The activation of *Houba* was further confirmed by eccDNA-specific PCR on the *Houba* circles (Fig. 5b–d).

## Discussion

In this study, we show the importance of Pol II in the repression of TE mobility in plants. By choosing the well-characterized heat inducible *ONSEN* retrotransposon, we were able to specifically address the role of Pol II in silencing transcriptionally active endogenous TEs in WT plants. Recent studies propose Pol II as the primary source for the production of TE-silencing signals that can then feed into the RNA silencing and DNA methylation pathways [15]. Our data strongly support these findings at two levels. First, we found that inhibition of Pol II activity reduced the degree of DNA methylation at *ONSEN*, demonstrating its distinct role in this process, and that Pol II also contributes to reinforcing silencing at the genome-wide level, primarily in the CHH but also in the CHG context. Second, our finding that DCL enzymes are sufficient to process the silencing signal produced by Pol II suggest that Pol II acts at very early steps in the TE silencing pathway by providing substrates to these enzymes. The observation that inhibition of Pol II in the *rdr6* background still further enhanced *ONSEN* accumulation after HS supports the notion that Pol II plays a central role in the previously proposed expression-dependent RdDM pathway [16].

Using mobilome sequencing we confirmed previous findings [2] that this approach is a powerful diagnostic tool to detect mobile retrotransposons: we detected highest levels of eccDNA of *ONSEN* in HS and drug-treated *Arabidopsis* seedlings and found new insertions in successive generations of these plants. Using the same approach on rice we were able to detect production of *Houba* eccDNA after drug treatments, suggesting that the progeny will then contain novel *Houba* insertions. This is still to be confirmed and may be hampered by the already very high *Houba* copy number present in the genome [27].

Our findings may indicate that Pol II is primarily involved in silencing young, recently active retrotransposons and perhaps to a lesser extent other tightly silenced TEs. Indeed, there are indications of very recent natural transposition events for *ONSEN* [28] and *Houba* [29] in the *Arabidopsis* and rice genomes, respectively. For instance, the annual temperature range has and may still contribute to contrasting *ONSEN* mobilization events in different *Arabidopsis* accessions [28]. *Houba* is the most abundant TE of the *copia* family in rice and has been active in the last 500,000 years [30].

Overall, our findings lead to the question of when plants lower their guard: under what conditions could Pol II be less effective in silencing TEs? Certain stresses that affect the cell cycle have been reported to lead to the inactivation of Pol II [31, 32]; this would provide a window of opportunity for TEs to be mobilized. Therefore, combined stresses that affect the cell cycle and activate TEs may lead to actual TE bursts under natural growth conditions. Interestingly, it has been reported that retrotransposon-derived short interspersed element (SINE) transcripts can inhibit Pol II activity [33]. This strongly suggests the presence of an ongoing arms race between retrotransposons and Pol II. Considering that almost all organisms analyzed so far have TEs [4] and RNA polymerases [34] and the reliance of TEs on host RNA polymerases, it may—from an evolutionary point of view—not come as a surprise that Pol II also has a function as an important regulator of retrotransposon activity. Strikingly, it has been shown in both *Saccharomyces cerevisiae* and *Drosophila melanogaster* that Pol II-dependent intra-element antisense transcription plays an important role in TE silencing [21, 35]. In addition, we observed a discrepancy in *ONSEN* transcript accumulation and measured ecDNA after HS in seedlings that were treated with zebularine only. This substantiates the notion that both the quantity and quality of transcripts affect regulation, reverse transcription and successful integration of retrotransposons. This is well in line with previous observations demonstrating that different TE-derived transcripts have distinct functions in the regulation of TE activity [36]. As a next step it will be of great interest to investigate if Pol II-dependent antisense transcription of TEs and subsequent dicer-dependent processing may be the key to solve “the chicken and the egg problem” of *de novo* silencing functional retrotransposons in eukaryotes.

Finally, our findings will allow future studies on the potential beneficial role TEs play in adaptation to stresses. Indeed, two recent studies point out the adaptive potential of retrotraonsposon and, more specifically, *ONSEN* copy number variation in natural accessions [28] and RdDM mutant backgrounds of *Arabidopsis* [37]. Upon mobilization, the heat-response elements in the LTRs of *ONSEN* [38] can create new gene regulatory networks responding to heat stress [11]. Therefore, it will now be of great interest to test if the *ONSEN* hc lines obtained in this study are better adapted to heat stress. This will allow us to test if retrotransposon-induced genetic and epigenetic changes more rapidly create beneficial alleles than would occur by random mutagenesis. Furthermore, the observation that HS did not lead to a stronger activation of *ONSEN* in hc lines compared to WT plants suggests that genome stability is not compromised in these lines. This result can be explained by at least two possible mechanisms: (i) the occurrence of insertions of inverted duplications of *ONSEN*, such as has been observed for the *Mu killer* locus in maize [39]—such insertions will lead to the production of double-stranded RNA feeding into gene silencing and thereby limit the activity of that TE; and (ii) balancing of TE activity and integrated copy number as has been described for *EVADE* in *Arabidopsis* [40]. In this case, when a certain TE copy number threshold is reached robust transcriptional gene silencing takes over, thereby limiting TE mobility and ensuring genome stability. The stability of new TE insertions is an important aspect in light of the future use of TEs in crop breeding and trait stability.

## Conclusions

TEs are important contributors to genome evolution. The ability to mobilize them in plants and possibly in other eukaryotes in a controlled manner with straightforward drug application, as shown here, opens the possibility to study their importance in inducing genetic and epigenetic changes resulting from external stimuli. Because the induced transposition of *ONSEN* can efficiently produce developmental changes in *Arabidopsis*, it will be very interesting to test if specific stress-induced TE activation can be used for directed crop breeding for better stress tolerance in the near future.

## Methods

### Plant material

All *Arabidopsis* mutants used in this study (*nrpb2-3* [14], *nrpd1-3* [41], *rdr6* [42], *dcl2/3/4* triple mutant [43]) are in the Col-0 background. For *O. sativa japonica*, the cultivar Nipponbare was used.

### Growth conditions

Prior to germination, *Arabidopsis* seeds were stratified for 2 days at 4 °C. Before and during stress treatments plants were grown under controlled conditions in a Sanyo MLR-350 growth chamber on solid ½ MS medium (1% sucrose, 0.5% Phytagel (Sigma), pH 5.8) under long day conditions (16 h light) at 24 °C (day) and 22 °C (night) (*Arabidopsis*) and 12 h at 28 °C (day) and 27 °C (night) (*O. sativa*).

To analyze successive generations, seedlings were transferred to soil and grown under long day conditions (16 h light) at 24 °C (day) and 22 °C (night) (*Arabidopsis)* in a Sanyo MLR-350 growth chamber until seed maturity.

For phenotyping, *Arabidopsis* plants were grown under long day conditions (16 h light) at 24 °C (day) and 22 °C (night) and short day conditions (10 h light) at 21 °C (day) and 18 °C (night).

### Stress and chemical treatments

Surface sterilized seeds of *Arabidopsis* and *O. sativa* were germinated and grown on solid ½ MS medium that was supplemented with sterile filtered zebularine (Sigma; stock, 5 mg/ml in DMSO), α-amanitin (Sigma; stock, 1 mg/ml in water) or a combination of both chemicals. Control stresses (6 °C for 24 h followed by control conditions for 24 h, CS) and heat stresses (6 °C for 24 h followed by 37 °C for 24 h, HS) of *Arabidopsis* seedlings were conducted as described previously [11].

### DNA analysis

For qPCR and prior to digestions, total DNA from *Arabidopsis* plants was extracted with the DNeasy Plant Mini Kit (Qiagen) following the manufacturer’s recommendations. For the qPCRs to measure the *ONSEN* copy number following HS and chemical treatments the aerial parts of at least ten *Arabidopsis* plants per replicate were pooled prior to DNA extraction. To track *ONSEN* copy numbers in the S1–3 generations of controls (only HS) and hc lines (HS + A&Z treatment) DNA from true leaves was extracted. For the estimation of the *ONSEN* transposition frequency, total DNA of pools consisting of at least eight seedlings of the progeny of HS + A&Z-treated plants was isolated. The DNA concentration was measured with a Qubit Fluorometer (Thermo Fisher Scientific). The copy numbers of *ONSEN* were determined with qPCRs on total DNA using a TaqMan master mix (Life Technologies) in a final volume of 10 μl in the Light-Cycler 480 (Roche). *ACTIN2* (*AT3G18780*) was used to normalize DNA levels. Primer sequences are given in Additional file 1: Table S1.

For the mobilome-seq analysis total DNA from the pooled aerial parts of three 10-day-old *O. sativa* seedlings was extracted as previously reported [44]. Genomic DNA (5 μg) for each sample was purified using a Geneclean kit (MPBio, USA) according to the manufacturer’s instructions. ecDNA was isolated from the GeneClean product using PlasmidSafe DNase (Epicentre, USA) according to the manufacturer’s instructions, except that the 37 °C incubation was performed for 17 h. DNA samples were precipitated by adding 0.1 volume of 3 M sodium acetate (pH 5.2), 2.5 volumes of ethanol and 1 μl of glycogen (Fisher, USA) and incubating overnight at −20 °C. The precipitated circular DNA was amplified by random rolling circle amplification using the Illustra TempliPhi kit (GE Healthcare, USA) according to the manufacturer’s instructions except that the incubation was performed for 65 h at 28 °C. The DNA concentration was determined using the DNA PicoGreen kit (Invitrogen, USA) using a LightCycler480 (Roche, USA). One nanogram of amplified ecDNA from each sample was used to prepare the libraries using the Nextera XT library kit (Illumina, USA) according to the manufacturer’s instructions. DNA quality and concentration were determined using a high sensitivity DNA Bioanalyzer chip (Agilent Technologies, USA). Samples were pooled and loaded onto a MiSeq platform (Illumina, USA) and 2 × 250-nucleotide paired-end sequencing was performed. Quality control of FASTQ files was done using the FastQC tool (version 0.10.1). To remove any read originating from organelle circular genomes, reads were mapped against the mitochondria and chloroplast genomes using the program Bowtie2 version 2.2.2 71 with --sensitive local mapping. Unmapped reads were mapped against the reference genome IRGSP1.0 (http://rgp.dna.affrc.go.jp/E/IRGSP/Build5/build5.html) using the following parameters: --sensitive local, -k 1. DNA from both mitochondria and chloroplast genomes integrated in nuclear genomes was masked (1,697,400 bp). The TE-containing regions cover 194,224,800 bp in *O. sativa*. Finally, the bam alignment files were normalized and compared using deeptools [45] and visualized with the Integrative Genomics Viewer (IGV) software (https://www.broadinstitute.org/igv/). Data from the mobilome analysis were submitted to GEO (accession number GSE90484).

The presence of circular *Houba* copies was tested by an inverse PCR on 7 ng of the rolling-circle amplified template that was also used for sequencing. A PCR specific to chloroplast DNA served as a loading control. PCR products were separated on a 1% agarose gel that was stained with a Midori Green Nucleic Acid Staining Solution (Nippon Genetics Europe). Primer sequences are given in Additional file 1: Table S1.

### Transposon display

The integration of additional copies of *ONSEN* into the genome of heat stressed and treated plants was ascertained by a simplified transposon display based on the GenomeWalker Universal kit (Clontech Laboratories), as previously described [11] with the following modifications: 300 ng of total DNA from adult plants in the S2 generation of heat stressed and A&Z-treated plants was extracted with a DNeasy Plant Mini Kit (QIAGEN) and digested with blunt cutter restriction enzyme *Dra*I (NEB). After purification with a High Pure PCR Product Purification Kit (Roche) digested DNA was ligated to the annealed GenWalkAdapters 1&2. The PCR was performed with the adaptor-specific primer AP1 and the *ONSEN*-specific primer Copia78 3′ LTR. The PCR products were separated on a 2% agarose gel that was stained with Midori Green. For primer sequence information, see Additional file 1: Table S1.

### Cloning, sequencing and genotyping of new insertions

To identify the genomic region of new *ONSEN* insertions, the PCR product of the transposon display was purified using a High Pure PCR Product Purification Kit (Roche), ligated into a pGEM-T vector (Promega) and transformed into *Escherichia coli*. After a blue white selection, positive clones were used for the insert amplification and sequencing (StarSEQ). The obtained sequences were analyzed with Geneious 8.2.1 and blasted against the *Arabidopsis* reference genome. The standard genotyping PCRs to prove novel *ONSEN* insertions were performed with combinations of the *ONSEN*-specific primer Copia78 3′ LTR and primers listed in Additional file 1: Table S1.

### RNA analysis and northern blotting

Total RNA from the aerial part of at least ten *Arabidopsis* seedlings was isolated using the TRI Reagent (Sigma) according to the manufacturer’s recommendations. RNA concentration was measured (Qubit RNA HS Assay Kit, Thermo Fisher) and 15 μg of RNA was separated on a denaturing 1.5% agarose gel, blotted on a Hybond-N^+^ (GE Healthcare) membrane and hybridized with 25 ng of a gel-purified and P^32^-labelled probe (Megaprime DNA Labelling System, GE Healthcare) specific to the full length *ONSEN* transcript (see Additional file 1: Table S1 for primer sequences). Northern blots were repeated in three independent experiments with the same results.

### Whole-genome DNA methylation analysis

Whole-genome bisulfite sequencing library preparation and DNA conversion were performed as previously reported [46]. Bisulphite read mapping and methylation value extraction were done on the *Arabidopsis* TAIR10 genome sequence using BSMAP v2.89 [47]. Following mapping of the reads the fold coverages of the genome for CS, CS + A, CS + Z and CS + A&Z were 13.4, 13.2, 18.4 and 16.3, respectively. Data from the bisulphite sequencing analysis have been submitted to GEO (accession number GSE99396).

### Statistics

Statistical analyses were performed with SigmaPlot (v. 11.0). Depending on the normality of the data, either an H-test or a one-way ANOVA was performed. The Student-Newman-Keuls method was used for multiple comparisons.

## Additional file

Additional file 1: Table S1. Table of all primers used in this study. **Figure S1.** Increase in *ONSEN* copy numbers in S1 pools of heat-stressed and Z-treated *nrpb2-3* plants. **Figure S2.** Detection of eccDNAs originating from ONSEN loci following heat stress and chemical treatments in *Arabidopsis*. **Figure S3.** Increase in ONSEN copy numbers in S1 pools of heat-stressed and A&Z-treated WT plants. **Figure S4.** Summary of confirmed novel *ONSEN* insertions in hc line 3. **Figure S5.** Stress-induced activation of *ONSEN* in the S3 generation after initial HS treatment. **Figure S6.** *Houba* forms LTR–LTR junction eccDNAs after combined A&Z treatment. (PDF 1660 kb)
