# Supplementary material for: Inhibition of RNA polymerase II allows controlled mobilisation of retrotransposons for plant breeding
Source: Genome Biol. 2017 Jul 7;18:134. doi: 10.1186/s13059-017-1265-4 (PMC5501947; doi:10.1186/s13059-017-1265-4)
Supplement: Additional file 1: Table S1. — Table of all primers used in this study. Figure S1. Increase in ONSEN copy numbers in S1 pools of heat-stressed and Z-treated nrpb2-3 plants. Figure S2. Detection of eccDNAs originating from ONSEN loci following heat stress and chemical treatments in Arabidopsis. Figure S3. Increase in ONSEN copy numbers in S1 pools of heat-stressed and A&Z-treated WT plants. Figure S4. Summary of confirmed novel ONSEN insertions in hc line 3. Figure S5. Stress-induced activation of ONSEN in the S3 generation after initial HS treatment. Figure S6. Houba forms LTR–LTR junction eccDNAs after combined A&Z treatment. (PDF 1660 kb) [file 13059_2017_1265_MOESM1_ESM.pdf]

Supplemental Table 1. Names purpose and sequences of primers used in this study.

Name	Sequence 5`->3`	Experiment	
GenWalkAdaptator1	GTAATACGACTCACTATAGGGCACGCGT GGTCGACGGCCCGGGCTGGT	Transposon Display	
GenWalkAdaptator2	(PHOS) ACCAGCCC (AMINO)		
AP1	GTAATACGACTCACTATAGGGC		
Copia78 3`LTR	AACACTTAAACACTTTCTCCA		
284 COPIA78-4219F_RT	CCACAAGAGGAACCAACGAA	qPCR	
285 COPIA78-4219R_RT	TTCGATCATGGAAGACCGG		
ONSEN probe	(FAM) AAG TCG GCA ATA GCT TTG GCG AAG A (BHQ1)		
ACT2_QT_F	TGCCAATCTACGAGGGTTTC		
ACT2_QT_R	TTACAATTTCCCGCTCTGCT		
ACT2_QT_probe	(JOE) TCCGTCTTGACCTTGCTGGACG (BHQ-1)		
OnsenFull_F	AAGTGGTATCAGAGCTTGAAGATCC	Northern blot	
OnsenFull_R	CAACACCCCCTCTTAAACTTGATTTTGC		
M13F	CGCCAGGGTTTTCCCAGTCACGAC	Cloning and sequencing	
M13R	TCACACAGGAAACAGCTATGAC		
houba_F2	ATCCTGGGAAGAACAAACCATTAA	PCR on circular rice TE and the chloroplast control	
houba_R2	GAGTTCGAGTACCTTAGCCATGGT		
Chloroplast cyc F	ACAACCACTGATGAAGGATT		
Chloroplast cyc R	AGAAAGAAAAGCAACGACTG		
Prove TED 2_20 R	ACCTAGCTCTGAGTGATGAA	# 1	Genotyping of novel *ONSEN* insertions
Prove TED4_27 F	TGGATATACACATTGGTTGCA	# 2	
Prove TED 2_19 F	GGAGAAAGCTGAAAACTTGG	# 3	
Prove TED4_30_rev	CTAGGTTGGTGACTGATGAG	# 4	
Prove TED 2_17 F	AAGAATGGGAGCAGCATTAA	# 5	
Prove3_2R	GCAGTACTATAACCGGGACT	# 6	
prove TED3_1 Fw	GAACTTTCCGTTGTTACCGG	# 7	
Prove TED3 F	ATGAGACAGGGAGCTTATCT	# 8	
Prove TED1 R	GGTGTGAACCGAACCTAAAT	# 9	
Prove TED 4_25 F	AAACACCAGAAATCTTTCGC	# 10	
tt6 fw	CACAGACCACAAGCATTTTT	TT6 gene	
tt6 rev	TGTCGATTTTCTTGGTGCTA		

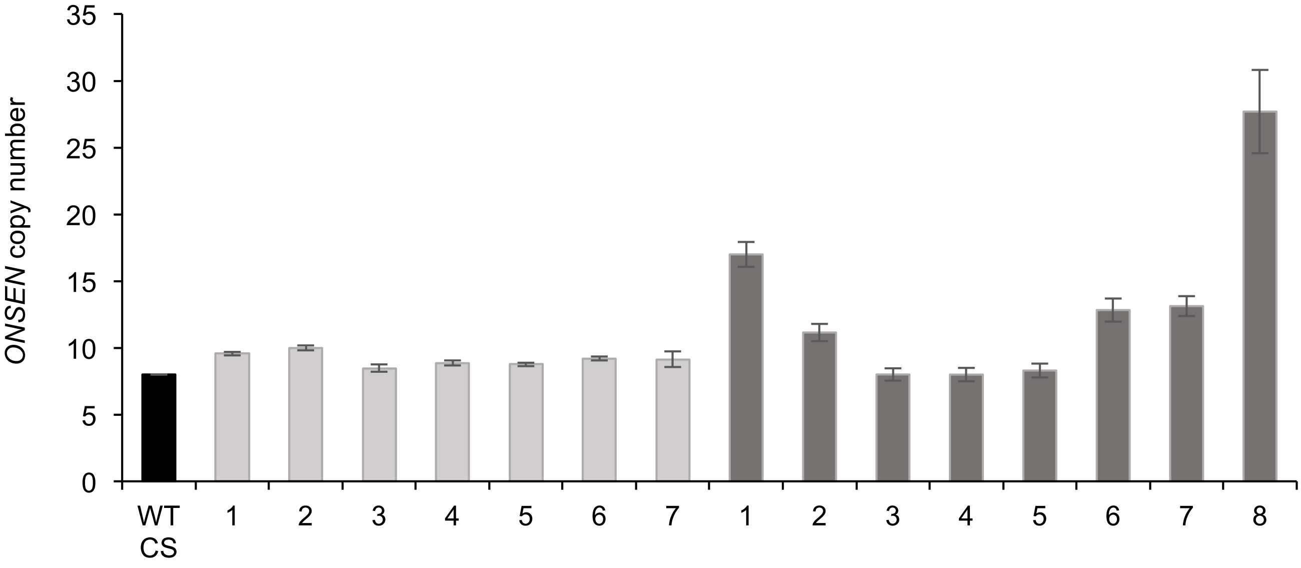

Fig. S1. Increase in *ONSEN* copy numbers in S1 pools of heat-stressed and Z-treated *nrpb2-3* plants. *ONSEN* copy number measured by qPCR in pooled seedlings of the S1-generation of heat stressed and zebularine-treated (10 µM) WT (light grey bars) and *nrpb2-3* plants (dark grey bars) that were grown under control conditions on soil relative to a control stressed WT-plant (black bar) (mean ± s.e.m., *n*=3 technical repetitions, all values relative to *ACTIN2).*

Fig. S1.

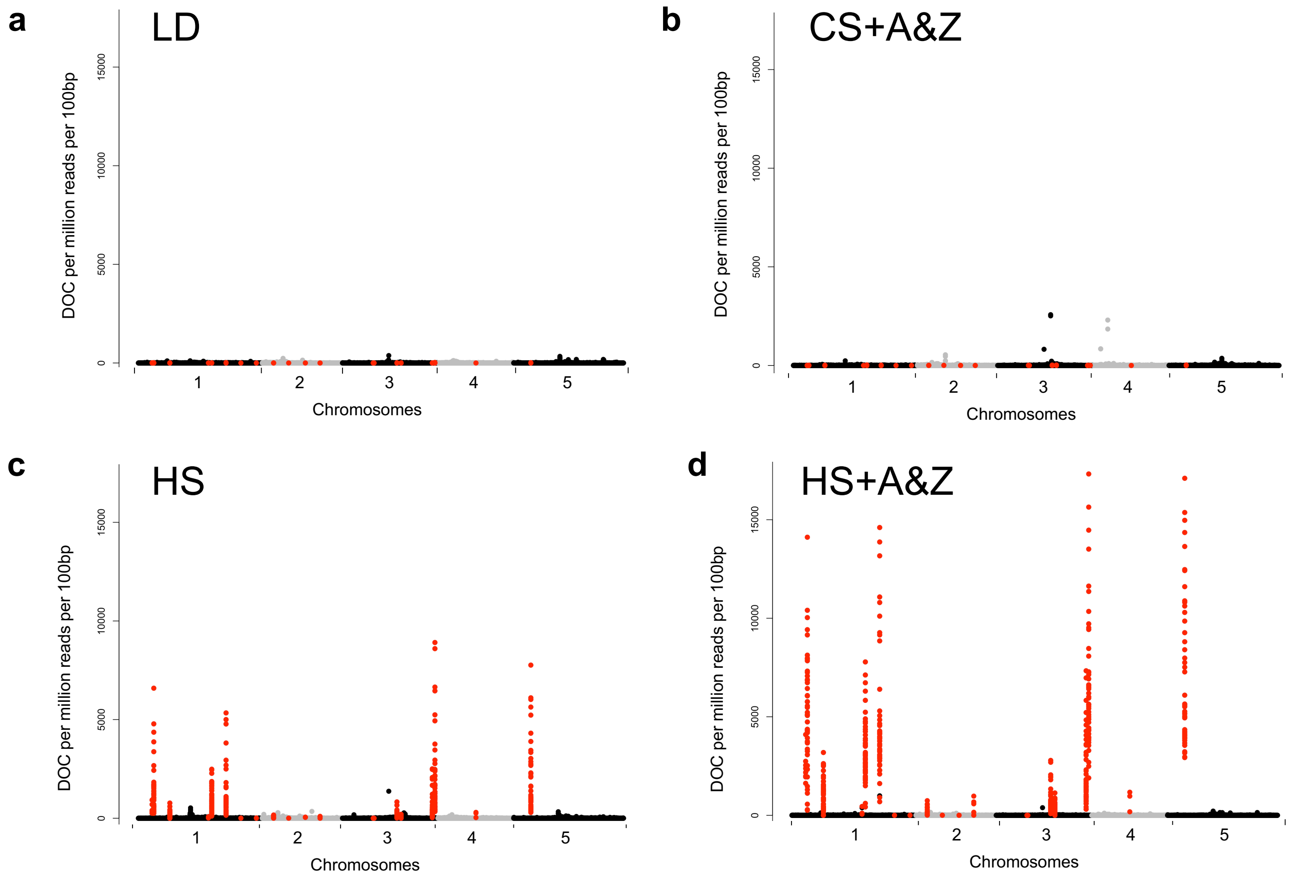

Fig. S2. Detection of eccDNAs originating from *ONSEN* loci following heat stress and chemical treatments in *Arabidopsis*. Abundance of reads from the mobilome-seq libraries of WT *Arabidopsis* plants mapping at TE-annotated loci from seedlings after: **a** growth under long day conditions (LD), **b** CS plus treatment with A (5 µg/ml) and Z (40 µM) (A&Z), **c** HS and **d** HS plus treatment with A&Z. Each dot represents the normalized coverage per million mapped reads per all TE-containing 100bp windows obtained after aligning the sequenced reads on the five chromosomes (black and grey circles). Red dots indicate the position of 100bp windows corresponding to *ONSEN* loci.

Fig. S2.

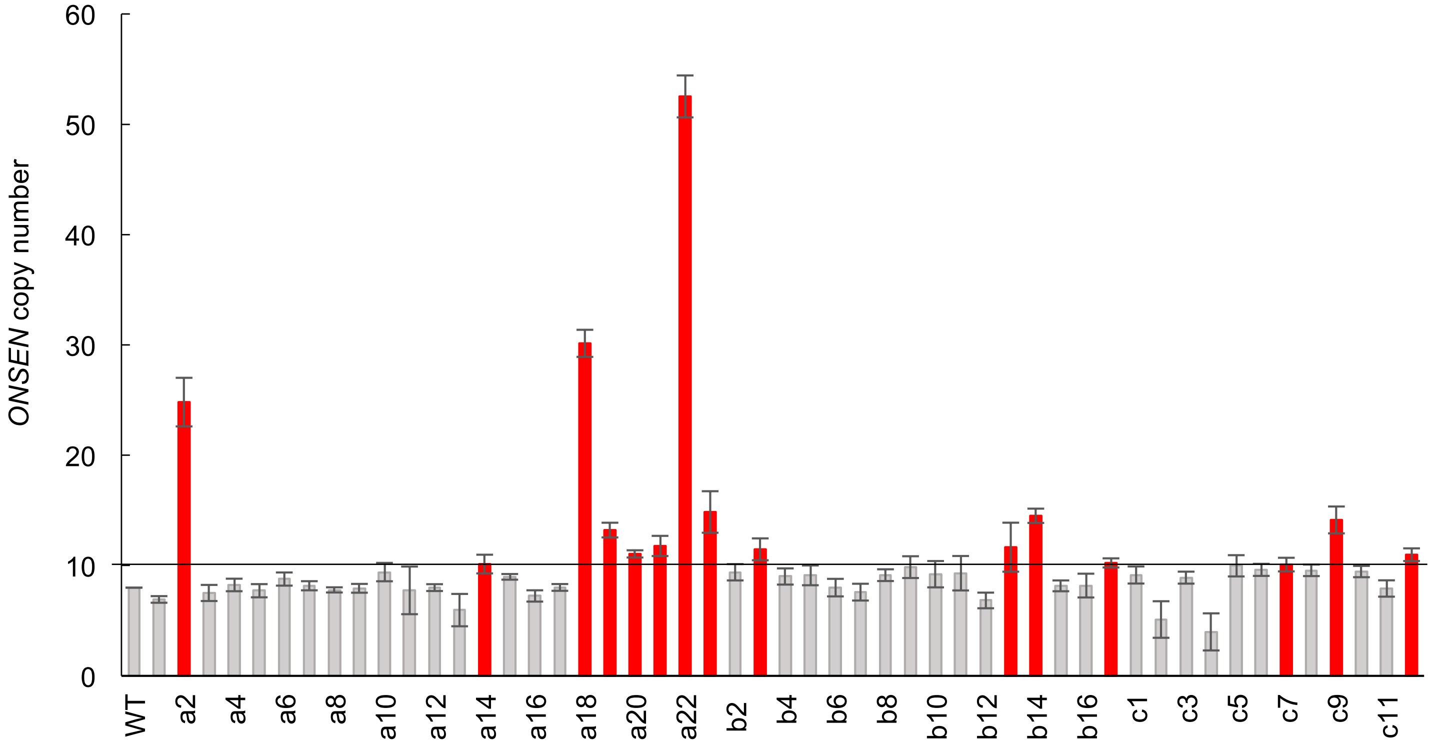

Fig. S3. Increase in *ONSEN* copy numbers in S1 pools of heat-stressed and A&Z-treated WT plants. Parental plants were heat stressed and treated in independent experiments (characters a-c) with a combination of A (5 µg/ml) and Z (40 µM). Pools with clearly increased *ONSEN*-copy numbers (>10) are marked in red. *ONSEN*-copy number measured by qPCR (mean ± s.e.m., n=3 technical repetitions, values relative to *ACTIN2*).

Fig. S3.

a

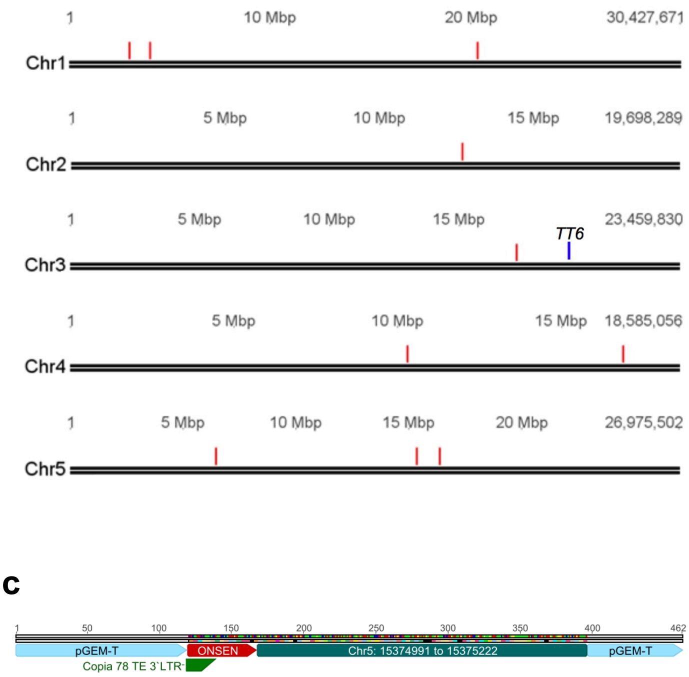

b

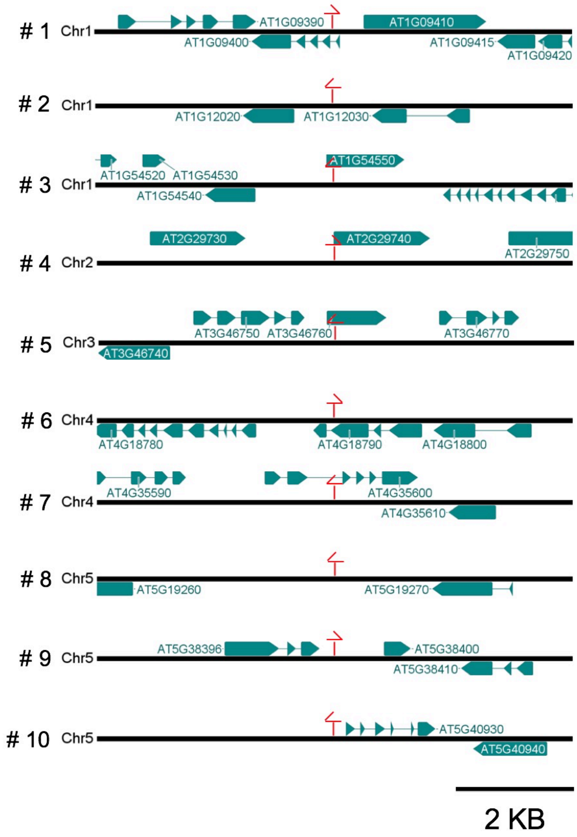

d

#	Chr.	Coordinates (5`)	#	Chr.	Coordinates (5`)
1	1	3034907	6	4	10318144
2	1	4063994	7	4	16897453
3	1	20369771	8	5	6483939
4	2	12706720	9	5	15374990
5	3	17222174	10	5	16404249

Fig. S4. Summary of confirmed novel *ONSEN* insertions in hc-line 3. a Overview of insertion sites shown in (**b**) (red bar) and the location of the *ONSEN* insertion in the *TT6*-gene depicted in Fig. 4 (blue bar) **b** Close-up of regions with new *ONSEN* insertions (red bar) in the S2 generation of a selected heat stressed and A (5 µg/ml) and Z (40 µM) treated WT plant (hc-line 3). Orientation of novel *ONSEN* insertions is indicated with red arrows. **c** A scheme to exemplify the annotation of sequences that lead to the identification of novel *ONSEN* insertion sites depicted in (**b**) shown for insertion # 9. Colors correspond to the pGEM-T vector (light blue) used for cloning, the *ONSEN*- 3`LTR (red), the Copia 78 TE 3`LTR primer (dark green) that was used for the preceding TE-Display PCR and the genomic region (turquoise) flanking the 3`LTR of the new *ONSEN* insertion. **d** Summary of coordinates (base 5` of insertion) of new *ONSEN* insertions shown in (**a**) and (**b**). Numbering corresponds to (**b**). Sequences of primers used to confirm new *ONSEN* insertions are given with the numbering corresponding to (**b**) in in Additional file 1: Table S1.

Fig. S4.

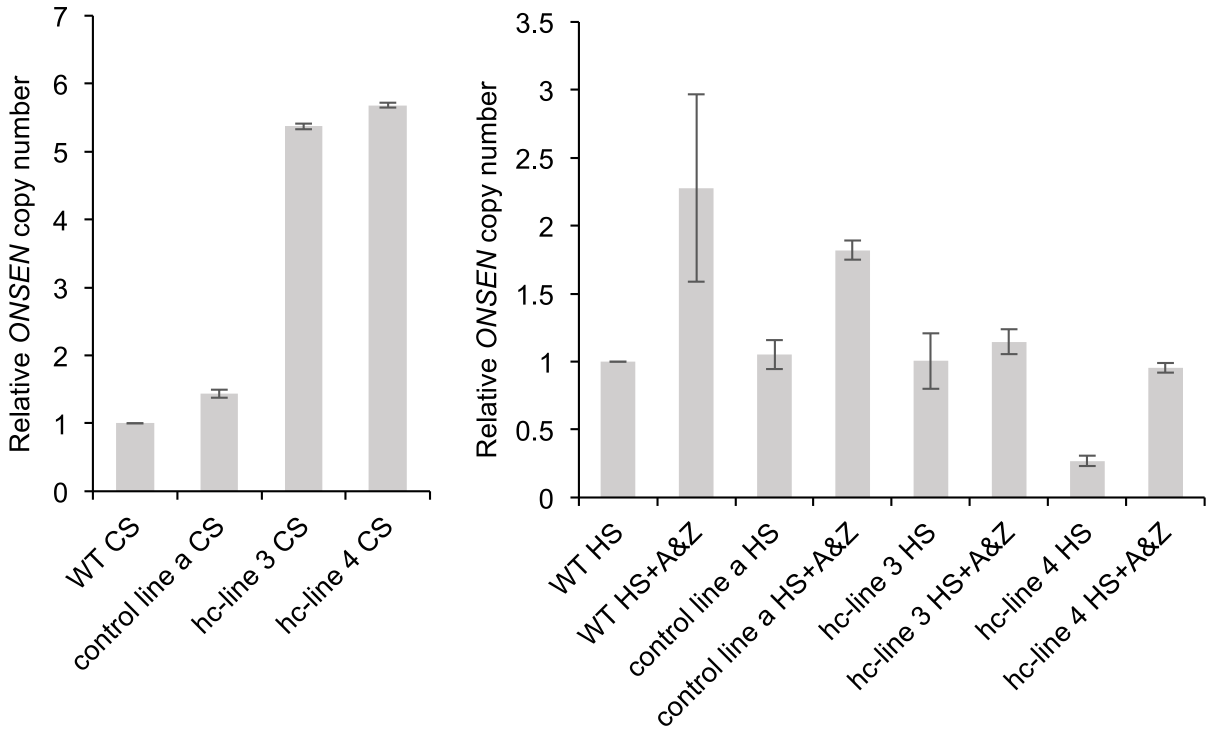

Fig. S5. Stress-induced activation of *ONSEN* in the S3 generation after initial HS-treatment. *ONSEN* copy number measured by qPCR directly after HS and HS plus treatments with α-amanitin (A, 5 µg/ml) and zebularine (Z, 40 µM) in seedlings of the WT, the control line a and the hc-lines 3 and 4. *ONSEN* copy number is shown relative to the WT HS (mean ± s.e.m., *n*= 3 biological repetitions, all values relative to *ACTIN2).*

Fig. S5.

```
READ    1    CTTTTAAGGAGGTCCCCCTCTCCTAGAATAAGCAAGGTGGTACTAAACTCCACATGCATG   60
             ||||||||||||||||||||||||||||||||||||||||||||||||||||||||||||
ref     290  CTTTTAAGGAGGTCCCCCTCTCCTAGAATAAGCAAGGTGGTACTAAACTCCACATGCATG   231

READ    61   CCATCCTATGAGGTGGGCTTTTGTGATTTTCCAAAGAATTAATCTTCGAGTGGGCTAAGG   120
             |||||| |||||||||||||||||||||||||||||||||||||||||||||||||||||
ref     230  CCATCCCATGAGGTGGGCTTTTGTGATTTTCCAAAGAATTAATCTTCGAGTGGGCTAAGG   171

READ    121  CCCATTCATTAATTCCAACAATCCCCCACATTGTTGAGATTATGGGCATATAATGATTTA   180
             ||||||||||||||||||||           |||||||||||||||||||||| ||||||
ref     170  CCCATTCATTAATTCCAACA-----------TGTTGAGATTATGGGCATATAAGGATTTA   122

READ    181  ATTTATTCCATAAATAAATCATGACATTACAGATGAAAACTAGCATGAACGCATCATTAG   240
             ||||||||||||||||||||||||||||||||||||||||||||||||||||||||||||
ref     121  ATTTATTCCATAAATAAATCATGACATTACAGATGAAAACTAGCATGAACGCATCATTAG   62

READ    241  ATCTACACATG   251
             |||||||||||
ref     61   ATCTACACATG   51
```

3’LTR 5’LTR

Fig. S6. *Houba* forms LTR-LTR junction eccDNAs when treated with the combination of A&Z. Alignment between a sequencing read resulting from the mobilome sequencing of A (5 µg/ml) and Z (40 µM) -treated plants (top) and an artificial junction corresponding to the 3’ part of the 3’ LTR (blue box) fused to the 5’ part of the 5’ LTR (yellow box).

Fig. S6.
